# STROKE

**DOI:** 10.1093/geroni/igz038.2126

**Published:** 2019-11-08

**Authors:** Monica C Serra

**Affiliations:** Emory University, Atlanta, Georgia, United States

## Abstract

Following stroke, the leading cause of disability in the United States, muscle alterations commonly occur. These alterations include gross atrophy and shift in fiber type, particularly in the paretic limb, which have lasting implications on gait ability. We will discuss the current evidence supporting a mechanism for these changes (i.e. physical inactivity, inflammation, oxidative stress, and decreased lower motor neuron activation) and how these factors differ between the paretic and non-paretic limbs. Further, we will discuss how various therapeutic exercise modalities can be used to modify or reverse skeletal muscle abnormalities and alter physical functioning post-stroke.

